# The co-conditioning of dis/ability and gender: An intersectionality study of Powerchair Hockey

**DOI:** 10.3389/fspor.2022.916070

**Published:** 2022-12-08

**Authors:** Laurent Paccaud

**Affiliations:** Health and Social Work Research Laboratory (LaReSS), Faculty of Social Work, University of Applied Sciences and Arts Western Switzerland (HETSL | HES-SO), Lausanne, Switzerland

**Keywords:** intersectionality, theory of sociology, dis/ability, gender, sport, Powerchair Hockey, sexism, ableism

## Abstract

This paper aims at initiating scholars to consider dis/ability as a category of analysis when doing intersectionality in sociology of sport. First, it introduces a conceptual framework that allows researchers to engage with the issue of the body and its physical and cognitive functions, as well as to address how the ability–disability system intersects with various other salient systems of oppression and privilege. I call this concept the *intersectional co-conditioning of dis/ability*, whereby experiences of dis/ability are fundamentally conditioned by (and also condition in return) other systems of difference and inequality. The framework provides scholars with theoretical tools that will help them to investigate body-related issues while avoiding the pitfall of essentializing dis/abilities. Second, this work offers an application of the abovementioned conceptual framework, focusing on the co-conditioning of dis/ability and gender. Based on a multi-sited ethnography of Powerchair Hockey in Switzerland, I investigate different aspects of this sport practiced by people living with so-called “severe” physical dis/abilities. The results highlight the tensions, contradictions and paradoxes that both male and female players face as they (re)negotiate their positions within the matrix of domination. This application demonstrates the explanatory power of considering the intersectional co-conditioning of dis/ability.

## Introduction

Since the beginning of the 1990s, intersectionality has gradually emerged as a highly relevant paradigm for critical feminist studies. Initially focusing on the intertwined and mutually constitutive aspects of gender and race (1), scholars gradually took into account other salient systems of difference and inequality, such as class, sexuality, nationality, which altogether constitute what Collins (2) calls a “matrix of domination.” Yet, to date, scholars who apply intersectionality still rarely address cognitive and physical dis/abilities^1^ and the related system of oppression [i.e., the “ability-disability system” (4)] in their analyses. Therefore, it is still common for scholars to work within an ableist paradigm of which they are often unaware and which they are likely to reproduce.

This issue is particularly salient in the context of sport sociology. Indeed, despite DePauw's (5) invitation 25 years ago to feminist scholars to address the lack of attention to the intersectionality between gender and dis/ability in sociology of sport, there is still little research that apprehends systems of difference and inequality in sport in regard to their intersection with the ability-disability system. Yet, physical and cognitive disabilities, abilities, and even hyper-abilities to enact a given behavior play a central role in the recognition of an individual's compliance with norms. Physical and cognitive dis/abilities to engage in socially expected attitudes and activities based on one's gender, race, nationality, ethnicity, class, and sexuality are an important dimension of the construction of differences and inequalities. Thus, the body and its cognitive and physical functions are central factors to be considered in sociology of sport. I name this phenomenon the *intersectional co-conditioning of dis/ability*, whereby experiences of dis/ability are fundamentally conditioned by (and also condition in return) other systems of difference and inequality. I therefore consider a systematic examination of the ability-disability system as a category of analysis while conducting intersectionality studies of sport to offer strong explanatory power. Such an approach will not only be useful for understanding the experiences of people with dis/abilities in sport but also for investigating sport in all its aspects.

In light of these preliminary comments, this paper pursues two objectives. First, it introduces a conceptual framework that allows researchers to engage with the issue of the body and its physical and cognitive functions and to understand how the ability-disability system intersects with various other salient systems of oppression and privilege, and more specifically with gender. Second, based on my doctoral dissertation on the sports careers of Powerchair Hockey (PCH) players, this paper offers an application of this conceptual framework. This application demonstrates the explanatory power of considering the ability-disability system while doing intersectionality in the sociology of sport.

In this article, in order to provide an in-depth analysis, I primarily focus on the intersectionality between dis/ability and gender. Indeed, PCH is a particularly interesting field to study the intersectional co-conditioning of dis/ability and gender because of the following two characteristics. First, in PCH, only people with so-called “severe” physical dis/abilities are eligible for participating in competitions, and most players are living with progressive genetic diseases. At the beginning of their sports career, some of them still have some mobility and strength in their upper body. Then, gradually, their physical impairments worsen. However, the game is regulated in a way that allows one to adapt their way of playing as their disease progresses; by changing the used material and their role on the field. Second, PCH is one of the few team sports in which women and men play on the same team. Physical dis/abilities, as well as the technologization of the body through the use of a powerchair, are considered by most of the actors of PCH as canceling the presumed physical advantages of men over women. Nevertheless, the experiences of Swiss PCH players should be understood as also being shaped by other salient systems of oppression, and the privileges they hold from belonging to “unmarked” social categories (6) as white, self-identified cis-heterosexuals living in a rich Western European country.

## Literature review: Studies on experiences of men and women with dis/abilities in sport

Studies of the interrelations between dis/ability and gender gradually emerged in the late 1980s (4, 7–9). Yet, it is only more recently that intersectionality specialists have begun to take dis/ability into account in their analyses^2^. Moreover, there is still little research on how dis/ability intersects with gender in the context of sport.

Feminist dis/ability scholarship^3^ show that women with dis/abilities are often “degendered” and “desexualized” as a result of (hetero)sexist and ableist norms. Sexism and ableism mutually contribute to stereotypical representations of women with dis/abilities as unfit sexual or romantic partners, inadequate mothers, passive and compliant, helpless victims, etc.—facilitating forms of exploitation (7, 14, 15). Only a few sociologists have studied the experiences of women with dis/abilities in sport (16–22). Most of this scholarship emphasizes that practicing sport allows women with dis/abilities to experience their bodies as sensitive and performant. Doing sport enables women to regain control of their bodies after an accident, which can lead to some forms of empowerment. Some scholars also take a more critical perspective on the experiences of women with dis/abilities in sport. Hargreaves [(23), p. 186–187] explains that unlike men with dis/abilities, “disabled women are affected by 'commodified anti-athletic stereotypes of femininity that do not make the sporting body into a physical capital to the same extent.” Female athletes with dis/abilities who embody the supercrip^4^ figure are less rewarded than male athletes with dis/abilities (22). Authors also shed light on the sexism that women with dis/abilities face within sport; explaining that, in the face of endemic sexism, some women with dis/abilities gather to practice solely among women and collectively resist ordinary sexism they experience (18, 20, 21, 25).

Although fewer in number, scholars^5^ have also begun to examine how living with dis/abilities affects men's gender' enacting. These authors show that men with dis/abilities lose some (but not all) of their “male privileges” through the process of “stigmatization” (27) related to society's perception of dis/abilities (28–30). They document how the acquirement of physical impairments constitutes a “biographical disruption” (31) for men (32, 33). Kvigne et al. (34) explain that men with dis/abling illnesses face significant structural barriers to enacting some of the characteristics or behaviors associated with “hegemonic masculinity” (35); physical strength, power, self-control, aggressiveness, competitiveness, risk-taking, denial of weaknesses, rejection of help, providing for financial needs (33, 36, 37). Therefore, men with dis/abilities are likely to be considered weak, dependent, passive, and pitiful, as well as asexual and ungendered (29, 38).

Research on the experiences of men with dis/abilities in sport shows that for men who have acquired physical dis/abilities in adulthood, committing to parasports, them to restore the social status associated with the embodiment of normative masculinity through the enacting of strength, risk-taking, and self-confidence (39–43). Other authors investigate the “role of sport in negotiating the dilemma of disabled masculinity” for men living with dis/abilities since an early age (44, 45). Paccaud and Marcellini (45) show that for a man living with “severe” physical dis/abilities since birth, commitment to powerchair sports allows him to be recognized by others as a man, without having to enact physical strength.

The body of research briefly presented above provides an important foundation from which to further investigate the intersectionality between dis/ability and gender in sport. Nevertheless, the following two gaps should be observed in future research. First, only a minority of the literature I present here refers directly to the concept of intersectionality; most of it focuses on sports experiences of men or women with dis/abilities, form either a dis/ability or a gender perspective. Thus, these authors usually theorize dis/ability and gender in isolation from each other, most often considering either dis/ability or gender as a “primary system of oppression” (46). In doing so, they engage in what Collins and Chepp (47) called “monocategorical thinking”. As a result, the mutual conditioning of dis/ability and gender is layered on after analyzing the dynamics of the gender order and the ability-disability order, respectively. However, as Hamilton [(48), p. 318] points out, “mutual conditioning does not capture what intersectionalities scholars describe as interlocking systems.” Second, by primarily focusing on the empowerment processes of people with dis/abilities through sport, some research has not fully captured the systems of difference and inequality that govern these social practices—the ability-disability system in particular. The purpose of this paper is to offer tools for scholars to overcome these gaps in future research.

## Theoretical framework: The intersectional co-conditioning of dis/ability

This section presents a conceptual framework that allows for integrating dis/ability as a category of analysis for intersectionality studies in sociology of sport.

Since the “biologization” of society that took place during the Age of Enlightenment, actors from the medical social world have defined body normalcy and have aimed to cure and redress bodies labeled as being pathological or abnormal (49). According to this medical perspective, “disability” is conceived as an individual-based problem that is a direct result of bodily impairments and the “deviance that [people with dis/abilities] maintain with respect to the social norm” [(50), p. 95]. The effort undertaken to reduce disability has focused on transforming individuals by treating them and bringing them back into the ability-disability norm.

Since the 1970s, the “social model of disability” has engaged in radical criticism of the medical model (3). The main conceptual innovation offered by the “social model of disability” is the reversing of the chain of causality behind dis/ability. The origin of dis/ability is no longer considered to be related to individuals but exclusively to the capitalist system that fails to meet their needs (51). Individuals are not dis/abled because of their impairments but because of the material and social barriers of the society in which they live (52). This allows for a de-essentialization of dis/ability, which is then considered “a matter of societal organization and social construction rather than of biology and the individual” [(53), p. 113].

The first limitation of the social model—long highlighted in theoretical debates—is that it eludes the issue of the body and its functions, which leads to the establishment of dualism between dis/ability (as a social disadvantage) and bodily impairments (54–56). This is all the more problematic for research in the sociology of sport, which investigates social phenomena in which the body plays a central role. Among the advocates of this critic, Kafer [(57), p. 7] refers to a political/relational model that offers a more embodied perspective. She explains:

“[T]he social model […] erases the lived realities of impairment; […] it overlooks the often-disabling effects of our bodies. People with chronic illness, pain, and fatigue have been among the most critical of this aspect of the social model, rightly noting that social and structural changes will do little to make one's joints stop aching or to alleviate back pain. Nor will changes in architecture and attitude heal diabetes or cancer or fatigue.”

The HDM-DCP is useful for conceptualizing the bodily experiences of people with dis/abilities in interaction with the environment (3). According to this model, people living with impairments, through their interactions with personal, community, and societal environment, can be in a social participation situation (i.e., the full realization of able-bodied life habits) in some environments or a dis/abling situation (i.e., the limitation of the fulfillment of abled-bodied life habits) in some others. Although the HDM-DPC can be criticized on other grounds^6^, the interactional way in which dis/ability is conceptualized is in line with intersectionality theories, which consider gender and race as dynamic and relational processes and not individual properties (6, 59).

A second critique of the social model that should be addressed to make it compliant with an intersectional approach is that it does not allow for an understanding of the normative system by which individuals are differentiated and hierarchized on the basis of their abilities and disabilities. Moreover, it problematizes dis/ability as a primary system of oppression. The contributions of critical dis/ability studies and crip theories^7^ are useful in resolving this two-sided pitfall. Indeed, several authors have questioned the “state of nature” of bodies, showing that bodies and their functions are always defined by socially constructed discourses and norms (49). Garland-Thomson [(4), p. 6] refers to an “ability-disability system” that “produces subjects by differentiating and marking bodies.” This system normalizes and benefits certain bodies and functions, which it sets up as standards—the ability-disability norms—and devaluates the bodies and functions that deviate from these standards. Non-normative bodies are then defined in terms of deficits, impairments, and disabilities (53). This system contributes to the production and legitimization of “an unequal distribution of resources, status, and power within a biased social and architectural environment” [(4), p. 5] to the advantage of those who can use their bodies in accordance with ability-disability norms. Following Garland-Thomson, some authors use the term “able-bodied,” which refers to the culturally shared definition of what it means to have normal physical and cognitive abilities (61). Other scholars draw parallels between the ability-disability system and other systems of difference and inequality. Over the past decade, the concept of ableism (62) has gradually gained popularity. Campbell [(62), p. 5] defines ableism as a “network of beliefs, processes and practices that produces a particular kind of self and body (the corporeal standard) that is projected as the perfect, species-typical and therefore essential and fully human.”

These amendments to the “social model of disability” allow for understanding the intertwining of different social forces and the intersectionality of relations of domination, which facilitates moving beyond an “additive model of oppression” to engage with an “interlocking model of oppression” [(2), p. 543].

To study the intersectional co-conditioning of dis/ability, the theoretical tools presented above must be compliant with gender theories usually used to apprehend the interrelations between dis/ability and gender; the same reasoning holds for other systems of difference and inequality.

If disability can only be conceived in relation to ability, the same reasoning should be applied to gender. Connell's work seems particularly well-suited to address this issue. Indeed, Connell demonstrates the inherently relational nature of masculinities and femininities, which require studying one in relation to the other to understand each of them (63). Furthermore, Connell (64) conceptualizes gender as a social “configuration of practices” produced and reproduced in different social contexts. Contemporary Western societies are structured by the generalized domination of men over women, through the power relations between each gender as well as within each gender (35). The domination of men over women is accomplished through a strategy that Connell refers to as “hegemonic masculinity.” According to Connell, both men and women do not form homogeneous social groups. Gender practices are enacted by individuals belonging to various social groups in terms of class, ethnicity, sexuality, and dis/ability, among others. Thus, Connell explains that, among men, certain social groups are in a position of subordination to hegemonic practices (e.g., men whose sexual practices deviate from heterosexuality). Moreover, other masculinities are “marginalized,” such as working-class masculinities, non-white masculinities, or “dis/abled masculinities” (26). She further distinguishes “complicit masculinities” from hegemonic masculinity, with the former being embodied by men who only partially behave in the manner prescribed by the hegemonic model but maintain it (passively), thus benefiting from patriarchy (35).

However, to ensure compliance with intersectionality, some adjustments to Connell's theoretical work are required (48). Indeed, Connell theorizes gender as an independent system of domination (i.e., the gender order). In contrast, as Hamilton et al. [(48), p. 319] note, intersectionality theories consider “axes in a matrix of domination as inextricably bound and mutually dependent form the start.” According to Collins (2), hegemonic masculinity goes beyond gender: “The organization of power in a given society is better understood as being shaped not by a single axis of social division, be it race or gender or class, but by many axes that work together and influence each other” [(65), p. 2]. Thus, although Connell's and Collins' works are convergent in many ways, an intersectionality approach is strained by Connell's idea that “the concentration of power in the hands of men leaves limited scope for [femininities] to construct institutionalized power relationships over another kind of femininity” [(66), p. 357]. For Collins (59), different femininities, like masculinities, are also constructed in relation to each other and hierarchized among themselves. Hamilton et al. [(48), p. 321] add that “all femininities may be subordinate to hegemonic masculinities, but some femininities play powerful roles in reproducing other forms of inequality.” According to these authors, both men and women can be oppressors and oppressed based on their positions vis-à-vis hegemonic expectations, which are shaped by multiple and interlocking systems of oppression. Thus, hegemonic femininity corresponds to a position of power (over other women and some men) in the matrix and women who embody it can derive significant benefits. Although approximating hegemonic femininity “requires women to defer to some men, motivations for doing so often involve pursuit of considerable individual and group benefits” (p. 326).

Having outlined the theoretical foundations for addressing the intersectional co-conditioning of dis/ability and gender, the next sections of this article examine the case of PCH in order to demonstrate how this concept operates in practice. Following the exposition of the methodology, I investigate five aspects of PCH: (1) the principles of sports classification; (2) the distribution and hierarchization of players' roles on the field; (3) players' athletic and aesthetic performances; (4) the unequal distribution of capital between players; and (5) the activism of players regarding ableism and sexism.

## Materials and methods

This paper is based on my doctoral thesis in which I aimed to understand how the commitment to a sports career in PCH forms and transforms the life trajectories of people with “severe” physical dis/abilities (58). Between 2015 and 2020, I conducted a multi-sited ethnography (67) of PCH in French- and German-speaking Switzerland, as an abled-bodied researcher in sports sciences interested in adapted physical activities. I conducted participant observations (550 h in total) at national and international competitions. I also observed practices in three different clubs. At the beginning of the survey, I was a complete outsider in the field. To gain acceptance among the PCH community, I assumed different roles and positions based on the demands of PCH actors (68). Thus, at times, I attended these activities as a discreet observer, at other times I helped set up the sports material, and on other occasions, I assumed the role of a caregiver for some players. I recorded all my observations in a field journal and took many photos and videos of the observed practices. In addition, I conducted a questionnaire survey to collect socio-demographic data on all players in Switzerland. A total of 99 players out of a total of 115 answered the questionnaire. I completed these data by collecting numerous documents (successive regulations of the practice, archives of some clubs and the Swiss Powerchair Hockey Committee, players' classification files, etc.). Finally, I carried out a series of 11 case studies with players (six men and five women), to understand their life trajectories.

The methodology for studying the life course of PCH players involved three data collection tools. First, I conducted a “life-course interview” (69). The initial assignment of the interviews consisted of the following question: “Could you explain how you became involved in Powerchair Hockey?”. When necessary, follow-up questions were then asked to investigate the other life trajectories of the interviewees (family, school, work, housing, insurance, medical, etc.). A few weeks later, I conducted a seven-day immersion in the life of each participant, where I moved through and interacted with their material and social environment (70) to capture their commitment to sport, as well as their various other commitments and daily habits. On the last day of immersion, I concluded with a “photo elicitation interview” (71), during which the interviewees were asked about twenty pictures they chose as best representing their different life phases (72). The interviews lasted from 1 h 50 min to 4 h 50 min.

I based my analysis on an inductive approach and performed a thematic analysis (73) of the data set. I paid particular attention to the “systems of symbolic opposition” (74) that emerged in the interviewee's discourses to uncover the meanings they give to their various experiences, status, and positions over time. Then, I modeled the “objectivable dimensions” of the various life trajectories of each player [(58), p. 115–119], and conducted a comparative analysis of their life courses.

Throughout the research protocol, I applied principles of relational ethics (75) in an effort to recognize and value mutual respect, dignity, and social relationships with participants. I have built research relationships with participants in a collaboration maintained from the beginning of data collection until publication. Participants were given the opportunity to examine and comment on the results of my analyses.

## Results

### Principles of classification in Powerchair Hockey

Since the beginning of modern sports, the classification of athletes has been an important concern in sport. From an institutional perspective, this dispositive (76) aims to establish fairness within competitions as well as elements of uncertainty in the result. To govern (and create) differences between athletes, categories of age, weight, and sex have been introduced based on the observation and measurement of biological differences in the body (77). Within able-bodied sports, these forms of classification are now mostly established and recognized as “natural”.

Throughout its history, PCH has always been played in competition within a unique category. The defining of the contours of this category does not follow the same principles as those of able-bodied sports, and the definition of this category has also shifted over time (58, 78).

Since 2016, the following regulation has applied. To be eligible, a player is required to: (1) Meet one of the following impairment types (impaired muscle power, impaired passive range of motion, or impaired coordination); (2) have a maximum of 4,5 points by adding the results of both arm and trunk MRC^8^ strength tests. Thus, the eligibility system selects the type of impairment and measures the function (strength) that is deemed to be the most decisive for athletic performances. In other words, it is not about assessing the biological differences between bodies, but rather assessing the impact of impairments for the considered sports tasks.

Although all eligible players play in the same and unique category, a classification system has been implemented to ensure fairness between players as well as between teams. A commission is in charge of assessing the degree of impact of the impairments in practice. Based on this assessment, each player is given a certain number of points. Players who have a great deal of strength and mobility in their arms and trunk and fine motor skills in the hands, who can powerfully shoot the ball, bend forward to reach the ball, and operate the joystick of their powerchair with precision, are scored with 4.5 points. On the other hand, players who cannot move their arms, can hardly turn their head, cannot shoot the ball other than with a cross fixed to the front of the powerchair (called “T-stick”) and who have a limited range of vision in a static position are scored 0.5 points. At competitions, the combined points of all five team members on the field cannot exceed a total of 12 points, which requires teams to be composed of players with a variety of functional profiles. In addition, at least two T-stick players per team must be on the field at all times. This dispositive allows the constitution of teams that are “equivalent” from a functional perspective. It also ensures a place in the game for those most impacted by physical impairments in practice. Thus, depending on the degree of physical dis/abilities, as their disease progresses and physical impairments worsen, players can adapt the way of playing while remaining indispensable to their team.

Among those involved in PCH (players, referees, coaches, spectators, caregivers), there is a consensus on the principle of excluding people whose dis/abilities are “not severe enough”^9^. This consensus builds on two agonistic principles.

First, there are identity- and community-based dynamics. Indeed, the doctor who developed the first version of this eligibility regulation explained that:

“The Duchenne, who are the majority of players, often already have a very short life expectancy, so it is very important to give them a place, to protect them from being excluded from their only sport.”^10^

David^11^, a player living with progressive muscular dystrophy, stated:

“I find it okay that people who don't use a powerchair are excluded. It's our sport, actually. At the limit, I would be okay with including people who have a progressive condition and who don't use a powerchair yet but will in the future.”

Moreover, this consensus builds on protectionist considerations, as Anna explained:

“This is important because otherwise, those who have the most severe physical disabilities are likely to be left out of the game.”

Thus, it is from a protectionist, medical, and identity-based logic, centered mainly around an etiological category—Duchenne muscular dystrophy—that PCH actors make sense of this eligibility system: to protect the players considered to be “the most disabled”.

Throughout the development of these regulations, it appears that a gender-based classification of players was never considered. When I raised this question to founding members of the International Committee for Powerchair Hockey, they were surprised that I would even question this possibility. Furthermore, in all of the International Committee for Powerchair Hockey Meetings Minutes, this issue is never mentioned, and when I raised this topic with the person in charge of implementing the current classification system, he said:

“Selective classification is important to allow everyone to participate reducing inequity to its minimum. For instance, if you do not separate men soccer players from women players, men would most likely always win. What sport needs is to have unpredictable competition, while allowing everyone to become world-class players. In Powerchair Hockey, impairment is the unit of classification.”

Among the players I met, the mixing of “sex categories” does not seem to be a matter of debate, but rather to be taken for granted^12^. This is indeed what Florian explained, whose opinion is shared by many players, both men and women:

“I haven't really thought about it. It's not like with able-bodied people, for whom, generally, men have more strength than women. Well, here, because of disability, there are often women who have more strength than men. Therefore, gender mixing has never been a problem. I never wondered about it, because I quickly understood why it was like that. Already by the fact that we have disabilities, well, the reason why it's separated in other sports is not relevant”.

PCH players subscribe to the belief that, among able-bodied people, men have higher physical abilities than women and men therefore have an advantage over women in the sport. However, they consider that, in PCH, the fact that all the players have significant physical dis/abilities invalidates the supposed physical advantages of men over women. Therefore, although they believe that “sex categories” are legitimate for able-bodied sports, gender-based classifications are not in PCH. This result confirms the findings of Richard et al. (21), who explain that, in Power-Soccer, physical impairments and the technologizing of bodies blurs the assumed physiological differences between women and men, thus legitimizing sports confrontations between the two.

The PCH classifications system operates as a dispositive of governmentality that categorizes and produces differences among players in a different and less binary way than able-bodied sports institutions. This alternative way of producing differentiation triggers some actors of PCH to challenge some of their cultural beliefs on dis/ability and gender. The following field journal fragment, which recounts exchanges I had with a classifier, provides a good illustration of this phenomenon:

While I am watching a game from the bleachers next to a classifier, she comments on the way a player she has just classified is playing. “See, it doesn't look like it, but we assigned Melina to class 4. If you look at her, she seems fragile, thin, girly, and... with her breathing apparatus on... If you compare her to Joshua, you would think that he would have a higher class than her. You know, he looks tough, a strong boy! But he was assigned class 3, so lower than Melina. First, I couldn't believe it. I never would have expected that.”

This fragment demonstrates the extent to which the classification system challenges the cultural beliefs of the classifier, who is herself able-bodied. The way she usually makes sense of dis/ability and gender properties of a person conflicts with what she observes. The ableist and sexist stereotypes, related to the appearance of bodies (i.e., women being naturally weaker, more fragile than men) are shattered, which leads her to re-evaluate her way of hierarchizing the players. The classification system operates as a certification process, not of the appearance of the body, but rather of the physical dis/abilities in situation, while also opening up rooms for challenging the ideology of a binary gender and the traditional organizational arrangement between masculinity and femininity in the social world of sport.

In the social world of able-bodied sport, the naturalized separation of women and men into two groups participates in the construction of men and women as two distinct and mutually homogeneous categories and contributes to the subordination of women as a group [(20), p. 110]. In PCH, through the innovating way in which differentiation on the basis of dis/abilities is performed, it is the absence of gender bi-categorization that PCH actors consider natural, which provides a clear example of the intersectional co-conditioning of dis/ability.

### Principles for the assignment and hierarchization of roles within a team

In PCH, within a team, each person has a specific role and mission. Players are divided into two main groups: those whose physical dis/abilities are such that they can handle the cross with an upper limb (called the “cross players”) and those whose physical dis/abilities are such that they cannot handle the cross with an upper limb and who play with a T-stick (called the “T-stick players”). Depending on this, players do not accomplish the same work on the field. The division of labor of players within the teams is further refined according to strength, physical dis/ability to pilot the powerchair as well as physical dis/ability to turn the head.

Depending on their role, players are not expected to develop and perform the same physical, technical, and strategical skills. According to Swiss national teams coaches' evaluation grid of players' skills, cross players should be able to “complete actions almost without error and with efficiency”, “hold and pass the ball without error”, as well as “have an optimal control of the ball”. T-stick players should be able to “block on the right opponent”, “execute correct, efficient blocking and carry it through to the end of the action”, as well as “mark, constrain and disturb almost any opponent”.

Throughout my observations, I did not notice any gender-based distribution of players' roles and missions on the field. Moreover, various coaches and players claimed that there is no gender bias in the distribution of labor on the field. A coach of the national team explained:

“Of course, women and men can have the same roles on the field. As a coach, I just look at the physical abilities, what they can do physically.”

Florian, a player on the national team, added:

“It doesn't appear to me that there is a difference. There are women and men who play in all positions. As striker or defender. No, I really don't think gender plays any role, it's more about the physicality.”

In PCH, everybody seems to agree that all the roles are essential to reach the main collective objective (i.e., winning). Nevertheless, power relations and the “hierarchical imaginary of the bodies” (77) that this division of labor contributes to creating, transforming, and/or reinforcing deserve to be further investigated. Indeed, analysis of discourses of people involved in PCH about the roles of cross players vs. the role of T-stick players highlights a hierarchy between the two; the ability-disability system and gender appearing to mutually build and reinforce this hierarchy. As an official explained, “a cross player is precious for a team. We are always looking for cross players”. Throughout their sports career and as their disease evolves, the majority of players transition from playing with a cross to playing with a T-stick. Thus, PCH clubs are constantly looking for new cross players. Conversely, they do not seem to actively recruit T-stick players. As a result, players who have a higher volume of physical abilities are particularly valued because of their rarity. Moreover, the work done by T-stick players is often invisiblized and is not recognized to the same extent as the work accomplished by cross players. For example, until very recently, there were no prizes or awards for the best T-stick players in competitions, whereas, as in able-bodied team sports, the best scorers were almost systematically rewarded. Furthermore, gender mutually shapes the social value of these two roles and the position that players hold in the matrix of domination is related to it. Indeed, the analysis sheds light on a normative system that values cross players to the detriment of T-stick players; the first category being considered related to masculinity, and the second to femininity. In that sense, ableism and sexism intertwine and mutually reinforce the hierarchization of bodies. This can be seen in the following field journal fragment:

Between two games, a coach and the mothers of some players comment on the fact that Thomas is playing with a T-stick for the first time in competition. The coach explains that this transition from playing with a cross to playing with a T-stick is a path that is often quite difficult for players: “especially for men”. A mother asks him why. He replies: “Well, I don't want to generalize, but guys want to be strikers, to be in the spotlight, so maybe it's a little less fun for them with a T-stick. And girls... for girls it's different, maybe it's more in their nature to stay a little bit more behind, to be at the disposal of the team.” His answer seems to convince the group. One of the mothers adds: “Yes, now that I think about it, Céline, who is a fighter, well, she has well accepted the T-stick”.

This result partly confirms the findings of Deal (81), Wickman (82, 83), and Berger (41) on other dis/ability sports. These scholars pointed out the existence of a hierarchy among athletes with dis/abilities: those with acquired physical impairments and with relatively minor dis/abilities stand at the top of the hierarchy, whereas those with congenital impairments and/or “severe” dis/abilities stand at the bottom of it. As in PCH, the athletes these authors met both activated and reproduced the conceptions of body normalcy that contribute to their oppression. However, applying an intersectional analysis demonstrates that the hierarchization of roles in PCH is not only shaped by the ability-disability system but is rather interlockingly determined by multiple systems of differences and inequality. Although my focus here is on dis/ability and gender, analyses show that players' class and migration background also play a role in the division of labor and the value attributed to the different roles [(58), p. 284–301].

### Undifferentiable athletic performances, yet different aesthetic performances between men and women

After more than 5 years of *in situ* observation, and after analyzing numerous videos of PCH games, I am still not even close to identifying any significant differences between the way men and women perform athletically in PCH. Indeed, it is very difficult, if not impossible to differentiate between the way men and women with similar degree and type of dis/abilities learn and perform the physical, technical, and strategical skills necessary to play PCH. Moreover, all the players with several years of practice demonstrate a strong will to take risks and overcome their limits, displaying perseverance, courage, and resistance to pain. To summarize, it can be said that both male and female players embody, albeit to different extents, the figure of the supercrip athlete.

Male players I interviewed noted, sometimes with surprise, that there is no difference between the way women and men play PCH. For example, Nicolas explained:

“Training sessions are the same for everybody. We train hard, we repeat the exercises, and we improve. In competition, men don't take more risks nor are better at holding duels or anything like that. It surprised me at first, to see women playing exactly the same way as men.”

Yet, some female players I interviewed explained that to make their way within the team, they adopt certain behaviors they associate with masculinity while distancing themselves from certain behaviors they associate with femininity. Maria explained:

“Sometimes, I guess I just go a tad harder. For example, when it's a duel, especially when it's a guy against me, I push a little harder. I impose myself. Because I'm a woman and there aren't many of us, I have to show off a little. I can't play like a girl, and well, I have more strength than most of them, so I can do it.”

In parallel, the majority of female players wish to appear as aesthetically feminine while playing PCH. As Maria, Monika and Céline told me, dressing in sports gear is experienced as a barrier to the way they want to “do gender” (84). At times, they clash with their coaches in an attempt to wear different outfits, which they consider to be more feminine, or to wear make-up and have a refined hairstyle, as the following field diary fragment illustrates:

Céline is the only female player present that day. She is also the only one not wearing a sports outfit. She wears makeup and designer clothes. On several occasions during the training session, players, the coach, and the mother of a player make jokes and negative comments about Céline's appearance, which is deemed inappropriate: “This is not a fashion show, this is sports”. Later, as Céline already left, they refer to her as a “diva”, a “miss” and a “princess” with a pejorative undertone.

In a social context mostly inhabited by men, the aesthetic performances of female players can be interpreted as a strategy to assert themselves as women. When the ideology of binary gender cannot be achieved and confirmed on the basis of differences in physical functions and athletic performances, the difference is created in another way—through aesthetic performance. However, the way they do gender is subject to disapproval, especially by some male coaches. It is indeed perceived by men (as well as some women) as incompatible with athletic performance.

The results confirm that the figure of the supercrip athlete crystallizes ableist values (24): overcoming one's limits, determination, independence, and achieving greatness. I also demonstrate that the local incarnation of this figure is mutually shaped by gender. Indeed, values associated with the supercrip figure intersect with configurations of gender practices associated with “hegemonic masculinity.” In this regard, for male PCH players, incarnating the supercrip figure contributes to being recognized as appropriately masculine. Nevertheless, in the context of PCH, the configuration of practices that embodies the local response to the perpetuation of patriarchy does not imply the physical superiority of men over women. If male PCH players try to approximate hegemonic masculinity, their efforts are not directed at achieving normative conformity in terms of physical abilities. In that regard, one can see forms of reconfiguration of power relations related to dis/abilities that create opportunities for challenging gender norms. For female players, embodying the figure of the supercrip athlete implies, to some extent, endorsing masculinity, or at least rejecting certain practices of hegemonic femininity. They must therefore make an extra effort to be perceived as appropriately feminine, as the values associated with hegemonic femininity in (hetero)sexist contexts are more related to ideas such as passivity and collaboration (4). They try, to the extent of their opportunities within the androcentric context of PCH, to assert certain femininity to be recognized as “proper” women. Although their margins of maneuver are limited, to some extent, female PCH players, by practicing a difficult sport involving agility, courage, resistance, control, risk-taking, self-improvement, and technical and strategic prowess, nevertheless challenge the social representations of people with dis/abilities as passive, deficient, fragile, and incompetent. They also challenge gender norms by constructing themselves as active subjects. However, when some of them do gender in a more normative way—which can be understood as an attempt to counteract processes of degendering and desexualization experienced by women with dis/abilities (4, 7, 53)—they are delegitimized within the group. Indeed, in the context of PCH, the approximating of hegemonic femininity comes at a cost, as it is deemed by a majority of PCH players to be incompatible with athletic performance.

To summarize, men who most accurately approximate the supercrip figure can rely on both gender and the ability-disability system to enhance their position in the matrix of domination. Those who do so participate in the oppression of both people with more severe dis/abilities and women, especially women with “severe” dis/abilities. Women who most closely approximate the supercrip figure face contradictory injunctions in negotiating their location in the matrix of domination. They have to find a critical compromise between the performance of athletic dis/ability and the performance of hegemonic femininity to reach a favorable location in the matrix of domination. If they perform either too well on one side or too well on the other, they may not gain as much benefit. Those who manage to find this balance participate in the oppression of people with more “severe” dis/abilities—among them especially women who resist hegemonic femininity. Thus, they participate in shoring up the existing order, which, although is to the advantage of their male counterparts, nevertheless offers them a favorable location in the matrix of domination.

### Gender biases regarding symbolic and social capital related to athletic performances

Throughout my research, I observed a significant number of players, both male and female, performing with prowess in terms of athletic skills. Discourses with players and coaches suggested that they consider “normal” the fact that, at a similar degree and type of physical dis/abilities, women demonstrate the same level of athletic skills as men. As Noah, a Swiss national team player, explained:

“It's clear that there are quite a few women who play at the same level as the men, even better for some. I'm in class 2.5 and so is Anna. If we have a duel between us, I don't know who would win.”

This suggests that the interactional configuration of PCH creates opportunities for changes in the way the intersectional co-conditioning of dis/ability and gender traditionally operates. At the interpersonal level, there is a form of acceptance of women's athletic skills and a reconsideration of the typical hierarchy between men and women in the world of sport.

The performance of high-level athletic skills participates in the embodiment of the figure of the supercrip athlete and allows for the accumulation of social and symbolic capital (85). Players who perform with a lower level of athletic skills are rewarded with less symbolic and social capital, which reinforces the ableist inequity between players. Men who have the highest level of athletic skills are often referred to by other players as examples to follow. Therefore, these players have an active role in the transmission of athletic skills to newcomers, as part of a “peer-to-peer emulation” process (86). Florian explained:

“When I started, the one who had the most technical skills, my model for me, was Christoph. He was my model, my idol. I set myself the goal to surpass him, and I did. Then, when Tim started, I was his model, and he eventually overtook me. The best is always above and young players are inspired by them.”

By contrast, I did not witness any process of peer-to-peer active transmission of athletic skills from female PCH players to novice players. Furthermore, throughout the interviews, a female player was never mentioned as a “role model” or an “idol” by any player. All players described to me role models as meeting the following two criteria. First, they have all been members of a national team during their careers. Secondly, they all have earned awards at high-level competitions. These awards, which are given by members of the National or International Powerchair Hockey Committee and attest to the players' athletic skills, are part of the institutional definition of good sportsmanship.

When men are awarded these distinctions, this generates unanimous admiration and respect from all those involved in PCH and increases their social and symbolic capital. When women who are members of national teams receive prestigious distinctions, it does not confer them the same capital as that of their male counterparts. On the contrary, their athletic skills are sometimes even delegitimized by some PCH players, as I observed at the European Championship in 2016—a competition at the end of which a female player was awarded the distinction of “best player”. The following excerpt of my field diary illustrates this point:

After the closing ceremony and the awarding of medals, several players, both male and female, comment on the fact that the “best player” award of the tournament was given to a Finnish female player. All of them expressed their disagreement with the awarding of this player. “It makes no sense, there are really better players than her. It's nonsense. You see, compared to Maas (a player who received this award several times and who was disqualified for unsportsmanship during the championship), there is no comparison, even with many others. She doesn't have the level.” Everyone agrees.

Thus, although men's athletic skills, when institutionally recognized, are never questioned by PCH players, women's athletic skills may be collectively delegitimized. When women achieve the normative expectations in terms of athletic skills, their athletic performances are not recognized to the same degree as those of men. As a result, the top of the hierarchy of PCH is for them much more difficult to access. Female players face barriers to embodying the role of peer-model. They are assigned the position of learning from men and rarely take on the role of passing on sport-related skills. In the social world of PCH, peer-to-peer emulation is gendered, and women are excluded from it, although the sport is mixed in terms of “sex category”. Additionally, sports participation offers less potential for empowerment for women than for men. This can be interpreted as a reminiscence of the androcentric norms in the social world of able-bodied sport (87, 88). “Norms that privilege characteristics associated with masculinity” [(89), p. 26] coupled with the cultural sexism that a large proportion of players have embodied, contribute to the devaluing of women's athletic performance. Although at the interpersonal level, the mixing of sex categories seems to create opportunities for changes in the interrelation of dis/ability and gender, at the community level, this is not the case. Indeed, in the absence of clear physical differences between female and male players, PCH actors find other ways to assert and embody the binary in ways that naturalize it. Richard et al. [(21), p. 12] explain that, in Power-Soccer, “if the players say that 'physically,' women and men are on an equal footing, in their speeches the same athletes, both women and men, establish behavioral and emotional differences between them.” In PCH, players rely on similar arguments to justify the hierarchy between male and female players. Thus, at the community level, the ideology of the gender binary persists, even in the absence of clear physical differences between female and male players.

### Powerchair Hockey players' activism against ableism and sexism

In PCH, I observed a collective action to fight against ableism suffered by the players in their daily life. Indeed, in a quasi-univocal sense, players conceive their commitment to this sport, which has been developed by and for them, as a means of fighting for access to participation in sports, and thus of having a so-called “normal” social participation. Anja, whose discourse is representative of this form of activism, explained:

“It is important for us to have this sport, our sport, because we created it, so we can also have access to sport. It's a question of equality. It's not because we are disabled that we shouldn't be able to play sports.”

Nevertheless, by embodying the figure of the supercrip athlete, PCH players—those who are most committed to competition in particular—simultaneously engage in producing difference and hierarchy within the social group of people with dis/abilities: those who manage to overcome their dis/abilities on one side and those who do not on the other. Nicolas' discourse is particularly illustrative of this process:

“It's important to show that even if you're disabled, you can achieve great goals. Not just staying at home and doing nothing. To show the world that you can accomplish great things.”

Yet, as already mentioned, players location in the matrix of domination is co-conditioned by whether or not they embody able-bodied gender norms.

Unlike resistance against ableism, female PCH players' resistance against sexism does not rise to a collective scale. This contrasts with the findings of scholars who studied other parasports mixed in terms of sex categories, such as Power-Soccer (21) and Wheelchair Basketball (20). These authors demonstrated that, in the face of sexism in parasport, some female athletes regrouped to form teams composed of a majority of women. This allows them to avoid having to fight on a permanent basis to “impose” themselves as women in a group of men. Yet, in PCH, female players, almost unanimously, rather want to stay “one of the few women” in a male-dominated team. Martina and Céline explained:

“I definitely don't want to be part of a team of women! If we were more, then there would be totally different group relationships. It would be more like a cat fight.”

“I wouldn't play in a women's team, not at all. I prefer competing with guys.”

In PCH, female players distance themselves from certain forms of sociability that they associate negatively with femininity, such as being jealous or gossiping. Confronted with femininity stereotypes that are negatively connoted and in which they do not recognize themselves, they do not identify themselves with sportswomen as a class. On the one hand, this represents a barrier to the building of a collective awareness of sexism. On the other hand, the attitudes of female PCH players concerning gender and feminism contribute to their integration into a male-dominated group. Moreover, female PCH players reported that “even if we wanted to get together to play powerchair hockey, it wouldn't be possible” (Anja). The main argument behind this is that, given their dis/abilities and the lake of human-based and material-based support, it would be very difficult, if not impossible for them to commute dozens or even hundreds of kilometers each week to practice among women. This result raises the following question. Throughout the discussions I had with female players, all of them but one expressed the desire to approximate hegemonic femininity. Yet, considering the sexism they face in PCH and the limited margins of maneuver they find to incarnate hegemonic femininity: why do they maintain their commitment to PCH?

My analysis revealed different reasons that account for why female PCH players maintain their commitment. First, some of the interviewees reported having had pleasant experiences of sport with their brothers and fathers before their PCH career. This suggests that women who engage in PCH may have internalized some “'inverted' gendered dispositions” (90) before committing to this sport. Indeed, Mennesson (90) explains that among the social factors that favor women's commitment to a career in a “male sport,” gender socialization plays a decisive role. Therefore, it is likely that among women with “severe” physical dis/abilities, only those who are particularly well-disposed to navigate this androcentric environment would engage in the first place.

Second, I hypothesize that if female PCH players maintain their commitment despite the sexism they face, it means that they are nevertheless receiving some benefits from practicing PCH. Female players reported having a lot of fun while participating in PCH; whether through the experience of driving fast with their sports powerchair, the experience of victory, the experience of traveling for competitions, or even the experience of belonging to a community. Thus, although athletic performances of women do not provide the same amount of capital as those of men, female players, by embodying sportswomanship, nevertheless acquire a certain symbolic and social capital, which allows them to negotiate a more favorable location in the matrix of domination. The capital they accumulate during their sports career may be sufficient for them to agree to maintain their commitment despite the constraints they face to do gender the way they would like to.

Third, all women who play PCH, in parallel to their sports career, strategically engage in other activities where they find more fertile ground for approximating hegemonic femininity, such as theater (Monika), writing (Anja), blogging (Céline) and beauty care (Maria). The following section of my field diary shows some of the gender-related and dis/ability-related implications of the interviewees' diversification of commitments in different social worlds.

I ask Monika why she continues playing PCH, despite the sexism she just described. She answers that it is important for her “balance” to practice sport: “so that I can get physical”. She explains that she joined a theater company 2 years ago. “Three ladies from the specialized institution where I live told me to come. So, three, it's already half of the women in the institution. I liked it. Now we are six of us. It's nice. For once, there are as many women as men. The dynamic is different from PCH: more mature and also other discussions, not always guy stuff. We are the ones who decide. Currently, I play the role of a fashionista.”

Like other female players, Monika has only limited possibilities to do gender as she would like to, both in the context of PCH and the specialized institution she lives in, which are two androcentric environments. Committing to a career in theater, in parallel to her sports career, reconfigures the possibilities she has for doing gender. The social world of theater seems more favorable to the gathering of women with dis/abilities. By joining together, these women manage to create margins of maneuver for enacting femininity. For Monika, as for other players, the multiplication of social worlds of inscription is the catalyst for gender self-determination. Thus, the position that players occupy in the matrix of domination, with respect to dis/ability and gender, mutually shapes the ways in which they can resist ableism and sexism, as well as the tactics they can deploy in an attempt to secure a more favorable location in the matrix.

## Conclusion

This paper provides a conceptual framework for integrating dis/ability as a category of analysis for intersectionality studies in the sociology of sport: the intersectional co-conditioning of dis/ability. This conceptual framework allows one to address body-related issues and to consider cognitive and physical functions while deconstructing the believed naturality of the differences between bodies. Thus, this conceptual framework brings perspectives to the sociology of sport to consider the body and its functions at the intersection of multiple systems of difference and inequality as ability-disability and gender, but also race, social class, or sexuality.

This article also provides an application of this conceptual framework *via* the case study of PCH in Switzerland. The findings partially confirm previous results uncovered by Sparkes et al. (39, 42, 43), Apelmo (20), and Richard et al. (21). In PCH, an innovative system of eligibility and classification based on the measurement of the impact of physical impairments in situation and the exclusion of those whose dis/abilities are “not severe enough” seem to legitimize the mixing of “sex categories”.

I demonstrate that in the context of PCH, the local variation of hegemonic masculinity does not rely on the believed physical domination of men over women. Thus, at the interpersonal level, this context allows for some subversion of the mutually constitutive relationship between dis/ability and the ideology of binary gender. Nevertheless, at the community level, this subversion only very partially challenges the power relations classically observed in the social world of able-bodied sport. The roles and positions held by players with the most “severe” dis/abilities are under-recognized and associated with femininity. Moreover, women's athletic performances are not recognized to the same extent as men's. Women are mostly excluded from the top of the sports hierarchy and certain valued roles such as the role of peer-model. Thus, ableism and sexism are interlocking, and they mutually reinforce the hierarchy between players. Therefore, in PCH, for men, embodying the figure of the supercrip athlete contributes to the process of normalization with regard to ability-disability and gender norms. Furthermore, women are confronted with contradictory injunctions. On the one side, they are required to erase signs of femininity to integrate within an androcentric environment and to be considered successful in sport. On the other side, female players are required to approximate hegemonic femininity to counteract the process of degendering and desexualization faced by women with dis/abilities.

The analysis of the intersectional co-conditioning of dis/ability in PCH shows how marginalized masculinities and marginalized femininities negotiate their locations in the matrix of domination. The results uncover the tactics that players, both male and female, use to draw benefits from the intersectional hierarchy, and they also highlight how those who can apply these tactics participate in the oppression of others and in shoring up the existing order. In light of the results presented in this article, one might extend the argument of Hamilton et al. (48), who explain that white—but also cis, straight, global north, and able-bodied—women who perform hegemonic femininity, despite it being advantageous to their male counterparts, nevertheless offers them a favorable location in the matrix of domination. Indeed, the analysis of intersectionality between dis/ability and gender in PCH suggests that all persons in a marginalized position may look to leverage the intersectional hierarchy in ways that grant them an advantage over certain others.

To further explore intersectionality in PCH in future research, it would be worthwhile to show how dis/ability and gender intersect with other salient systems of difference and inequality, such as race, class, or sexuality, among others. Indeed, these elements also frame in a significant way this sport and the experiences that one can have of it.

To conclude, I would like to emphasize that the heuristic potential of the suggested conceptual framework is not limited to the study of sports experiences of people with dis/abilities. On the contrary, I believe that it also holds an explanatory power to better understand sports practices of people socially defined as able-bodied as well as people with hyper-abilities. For instance, I perceive a strong heuristic potential in the application of this conceptual framework to the study of femininity tests and the principles of justifications of “sex categories” in abled-bodied sports. Thus, this paper is an invitation to fully engage in an intersectionality approach that considers dis/ability as central categories of analysis. This invitation is not only directed at researchers interested in studying experiences of people with dis/abilities in sport, but to all researchers doing sociological studies on sport.

## Data availability statement

The original contributions presented in the study are included in the article/supplementary files, further inquiries can be directed to the corresponding author/s.

## Author contributions

The author confirms being the sole contributor to this work and has approved it for publication.

## Footnotes

^1^In this paper, I use the terms “people with dis/abilities” and “people with impairments” in reference to the Human Development Model and Disability Creation Process (HDM-DCP) (3) and to imply that when we look at “people with impairments”, we should not only focus on their “disabilities” but also their many “abilities”. In their daily interactions, interviewees sometimes use identity-first language and sometimes person-first language without any consistency. For clarity, unless citing a direct quotation, I use person-first language.

^2^It should be noted that these authors have different takes on intersectionality theories.

^3^For more exhaustive syntheses of this literature see Hall (10), Mohamed and Shefer (11), Simplican (12), as well as Naples et al. (13).

^4^The concept of “supercrip” refers to people living with dis/abilities who “go beyond” and “overcome” their dis/abilities to achieve professional, athletic, and social success. While, from an ableist perspective, these accomplishments are considered to be grandiose, critical dis/ability studies and crip studies remind us that these achievements would be banalities of everyday life if they were not made complicated by an environment unsuitable for people with dis/abilities. Indeed, the narrative of the supercrip figure always strives to overcome and erase dis/ability, while invisibilizing the material and ideological structures that impede various achievements (24).

^5^For more exhaustive syntheses of this literature see Shuttleworth et al. (26).

^6^See Paccaud [(58), p. 46–50].

^7^For an overview of critical dis/ability studies and crip theories, see Meekosha and Shuttleworth (60).

^8^The MRC scale, which was developed by the Medical Research Council, is a gradation scale for muscle strength (79). The score of 0 corresponds to having no strength at all and the score of 5 corresponds to the strength expected from an able-bodied person.

^9^Those who are not eligible are encouraged to take on other roles such as coach, referee, caregiver, or supporter.

^11^Quotations have been translated either from French, German, Swiss-German dialects, or Dutch by myself.

^12^The names of the research participants quoted in this article have been changed.

^13^Yet, in 2017, among the 99 players who took part in my questionnaire survey (of a total of 115 existing Swiss players), there was a large majority of men: 82 male players to 17 female players. This wide disparity between “sex categories” has also been observed by Richard [(80), p. 281] in Power-Soccer, the main other team sport played with powerchairs. Richard explains that “for athletes, this is explained by the fact that potential female players may be turned off by the idea of playing a 'male' sport, Soccer.”
